# Prevalence of Working Memory Impairment in Drug Naive Schizophrenic Patients in a Tertiary Care Hospital

**DOI:** 10.31729/jnma.4642

**Published:** 2019-10-31

**Authors:** Bharat Kumar Goit, Jai Bahadur Khattri

**Affiliations:** 1 Department of Psychiatry, Janaki Medical College, Janakpur, Nepal; 2 Department of Psychiatry, Manipal College of Medical Sciences, Pokhara, Nepal

**Keywords:** *age of onset*, *memory*, *schizophrenia*

## Abstract

**Introduction:**

Schizophrenia is a mental disorder in which clear consciousness and the intellectual capacity are usually maintained, although certain cognitive deficits may evolve in the course of illness. Working memory impairment in schizophrenia affects the course, outcome, and quality of life of the patient significantly. The aim of the study was to find out the prevalence of working memory impairment in drug naive schizophrenic patients.

**Methods:**

This descriptive cross-sectional study was done in a tertiary care hospital, from June 2015 to December 2015 after taking ethical clearance from institutional review committee registration number 38970/062/063. Convenience sampling was done. The samples of 30 schizophrenic patients between the ages of 15 to 45 years were enrolled from the inpatient and outpatient unit of department according to inclusion and exclusion criteria. Data was collected and entry was done in Epi Info ver.7 point estimate at 95% Confidence Interval was calculated along with frequency and proportion for binary data.

**Results:**

The prevalence of working memory impairment was 26 (86.7%) in digit span forward test and 27 (90%) in digit span backward test. The mean age of patients was 27.5 years and the age of onset of schizophrenia was mainly in the age group between 16 to 25 years 16 (53.3%).

**Conclusions:**

These finding suggests high prevalence of working memory impairment in schizophrenic patients.

## INTRODUCTION

Schizophrenia is a mental disorder in which clear consciousness and the intellectual capacity are usually maintained, although certain cognitive deficits may evolve in the course of illness.^1^ Schizophrenia is prototype psychotic disorder and one of the most important illnesses in psychiatry, the expression of symptoms varies across patients and over time.^2^ The study conducted in the 14 countries for disability associated with physical and mental condition, active psychosis was ranked third.^3^

There is increasing evidence that working memory dysfunction is a core cognitive deficit in schizophrenia. Memory impairments are quite common and often moderate to severe in magnitude in schizophrenia.^4,5^

Working memory impairment in schizophrenia affects the course, outcome and quality of life of the patient significantly and thus needs adequate attention in the management.

The objective of the study is to study the prevalence of working memory impairment in the drug naïve schizophrenic patients.

## METHODS

This cross-sectional study was conducted at National Medical College, Birgunj, Nepal from June 2015 to December 2015. The ethical approval was taken from institutional review committee and consent was taken from all the patients. In case where the patient was not able to give consent, consent from family member was sought. Inclusion criteria for the participants in the study were drug naive patients between the ages of 15 to 45 years who were enrolled from the inpatient and outpatient psychiatric department and who had fulfilled the diagnostic criteria of schizophrenia according to ICD-10 criteria^1^ and the exclusion criteria was patients having any other co- morbid psychiatric and/or physical illness which affects cognitive functioning.

Semi structured proforma was used to obtain the socio-demographic characteristic of patient. The patients were assessed using digit span test for the test of memory from Wechsler Adult Intelligence Scale (WAIS).^6^

Convenience sampling was done and sample size was calculated using the formula,

n = Z^2^ × p × q/e^2^

n = (1,96)^2^ × 0.95 x 0.05/0.08^2^

   = 28.51

   = 29

where,

n = Sample size
Z = 1.96 at 95% confidence interval
p = prevalence, 95%
q = 1-p, 5%
e = margin of error= 8%

The total sample size calculated is 29. Data entry was done in Epi Info ver.7, point estimate at 95% CI was calculated along with frequency and proportion for binary data and analysis was done.

## RESULTS

The prevalence of working memory impairment was 26 (86.7%) in digit span forward test and 27 (90%) in digit span backward test. The mean age of patients was 27.5 years. Fourteen (46.7%) of the patients were between 16 to 25 years of age. Both male and female were equal in number in the sample.

The socio-demographic characteristics of the respondents are shown. The maximum respondents were unemployed and were Hindu religion followers (Table 1).

**Table 1:** Socio-demographic characteristics of the respondents.

Sociodemographic variables	n (%)
Age (in years)	
16-25	14(46.7)
26-35	11 (36.7)
36-45	5(16.7)
Gender	
Male	15(50)
Female	15(50)
Occupation	
Student	9 (30.0)
Employed	2(6.7)
Unemployed	11 (36.7)
Housewife	8 (26.7)
Religion	
Hindu	25 (83.3)
Buddhist	5(16.7)

The distribution of the age of onset of the respondents is shown. Sixteen (53.3%) schizophrenic patients had the onset of illness between 16 to 25 years of age. Eleven (36.7%) of the patients had onset at the age between 26 to 35 years and only 3 (10%) patients had onset between 36 to 45 years (Table 2).

**Table 2:** Distribution of patients according to age of onset of schizophrenia.

Age of onset (in years)	n (%)
16-25	16(53.3)
26-35	11 (36.7)
36-45	3(10.0)

Abnormal findings of digit span test of the schizophrenic patients is shown. The patients with schizophrenia had performed poorer both in digit span forward and digit span backward (Table 3).

**Table 3:** Impaired digit span findings of the patients.

Digit span test	n (%)
Digit span test (Forward)	26 (86.7)
Digit span test (Backward)	86.7 (90)

## DISCUSSION

The prevalence of working memory impairment was 86.7% in digit span forward test and 90% in digit span backward test. The mean age of patients with schizophrenia in the current study was 27.5 years and the majority of the patients (46.7%) were in the age group between 16 to 25 years. The mean age of patient according to one study attending psychiatry OPD in Kathmandu was 29.87 years and 80% of the patients were from the age group of 11–40 years.^7^ In another study at Kathmandu, the mean age of patient attending general psychiatry OPD was 27.28 years and the in the class interval categorization majority of patients falls in the age group between 15–34 years (72%).^8^ Many previous studies have consistently noted that the most of the patients seeking psychiatric services were younger, more than half coming from age group between 21 to 40 years.^9-11^ In the in-patient population, one study reported the mean age of the patients of 30.67 years and 58% of the patients coming from the age group between 21 to 40 years.^12^ The study in Nigeria found mean age of 34.81 years with 20-29 years being the most predominant age group.^13^

Similar to the other studies,^13,14^ this study also found that schizophrenia was equal in both genders. However, the other study conducted in Pokhara noted 61.9% of males and 38.1% of females.^15^ The study done in Bangladesh found that males suffers more as compared to females.^16^ Whereas, the another study done in Australia found more prevalence in female patients.^17^

In this study, the majority (36.7%) of the patients were unemployed followed by students (30.0%). This higher prevalence of unemployment indicates the socio occupational impairments seen in schizophrenia. The unemployment rates have been reported to reach almost 80% in simple cases and it is estimated that schizophrenia constitutes 10% of permanently disabled population.^18^ The unemployment rate in schizophrenia ranges from 36.9%,^13^ 51.2%,^14^ 52.4%^19^ to 86.7%^15^ in different studies.

Majority of the patients were Hindu (83.3%) followed by Buddhist (16.7%). Another study done in Nepal in the patients of Schizophrenia also found that majority of the respondents were Hindus (78.1%), followed by Buddhists (15.2%), Christians (3.8%) and Muslims (2.9%).^15^ This may be due to the fact that majority of Nepalese population follows Hindu religion.^20^

In this study the age of onset of schizophrenia was between 16 to 45 years. Out of this, the majority of the patients (53.3%) were in the age group between 16 to 25 years followed by 26 to 35 years (36%). This finding was also supported by the previous literature.^13^

Digit Span Test (forward and backward) from Wechsler Adult Intelligence Scale was used in the current study to measure working memory. The finding of the present study showed that there is high prevalence of working memory impairment in digit span forward (86.7%) and digit span backward (90.0%) test. The finding of this study was consistent with another study.^21^ The other studies also found that up to 98% of patients with schizophrenia perform poorly on cognitive tests than would be predicted by their parents’ education level. In several cognitive domains, the average cognitive impairment in schizophrenia can reach two standard deviations below the healthy control mean.^22,23^

Since this study was done in small sample size and convenient sampling was done, the findings of this study can’t be generalized, but the high prevalence of working memory impairment in Nepalese schizophrenic patients is of concern. The findings may have implication in understanding the nature of cognitive impairment in schizophrenia, in planning treatment strategy and in implementing a proper rehabilitation programme.

Hence, the further study with random sampling and larger sample size is recommended. Also in this study, only one domains namely, memory were considered for assessing cognitive dysfunction. Inclusion of other cognitive domains testing and neuroimaging techniques might provide new information.

## CONCLUSIONS

There is high prevalence of working memory impairment in schizophrenia. This domain should be taken into account in neuropsychological evaluation and efforts at remediation in the Nepalese context.

## Conflict of Interest:

None.
